# A Cross‐Sectional Virological and Sero‐Epidemiological Study of Exposures to Avian Influenza A(H5N1) and A(H9N2) Viruses in Live Bird Market Workers in Dhaka, Bangladesh

**DOI:** 10.1111/irv.70189

**Published:** 2025-11-16

**Authors:** Mahbubur Rahman, Timothy M. Uyeki, Malik Peiris, Jacqueline M. Cardwell, Patrick Nguipdop‐Djomo, Min Kim, A. S. M. Alamgir, A. K. M. Muraduzzaman, Sudipta Sarkar, Md Giasuddin, Md Ahasanul Hoque, Montse Torremorell, Mahmudur Rahman, Guillaume Fournié, Dirk U. Pfeiffer, Meerjady Sabrina Flora, Punam Mangtani

**Affiliations:** ^1^ Royal Veterinary College Hatfield UK; ^2^ Institute of Epidemiology Disease Control and Research Dhaka Bangladesh; ^3^ Influenza Division Centers for Disease Control and Prevention Atlanta Georgia USA; ^4^ The University of Hong Kong; ^5^ London School of Hygiene and Tropical Medicine London UK; ^6^ Bangladesh Livestock Research Institute (BLRI) Savar Bangladesh; ^7^ Chattogram Veterinary and Animal Sciences University (CVASU) Chattogram Bangladesh; ^8^ University of Minnesota College of Veterinary Medicine; ^9^ National Research Institute for Agriculture, Food and the Environment France; ^10^ City University of Hong Kong

**Keywords:** avian influenza, avian influenza A virus, Bangladesh, live bird market, seropositivity

## Abstract

**Background:**

Avian influenza A viruses (AIVs) are endemic among poultry in Bangladesh sold at live bird markets (LBMs). We assessed virologic and serologic evidence of exposure to AIVs among LBM workers.

**Methods:**

A cross‐sectional study recruited 702 randomly sampled workers from 42 LBMs in Dhaka, Bangladesh, during 2017. Nasal and throat swabs collected from workers and air samples from LBMs were tested for influenza A virus by RT‐PCR with positives subtyped for A(H5), A(H7), and A(H9). Baseline sera from 695 workers and follow‐up sera from 89 workers with influenza A positive respiratory specimens were tested by microneutralization assay for antibodies to A(H5N1) clade 2.3.2.1a and A(H9N2) G1 lineage viruses circulating in poultry. A seropositive result was defined as a neutralizing antibody titer ≥ 1:40.

**Results:**

Most LBM workers reported slaughtering (93.3%) and defeathering (84.5%) poultry. Ninety‐nine (14.1%) had ≥ 1 respiratory specimen that tested influenza A positive but negative for A(H1) and A(H3). Of these 99, subtyping identified 28 (28.3%) A(H9), 2 (2%) A(H5), 3 (3%) both A(H5) and A(H9), and 66 (66.7%) A (nonsubtypeable). Influenza A viruses were detected in air samples at 25 LBMs (59.5%), including A(H9) only in 10 LBMs (40%), A(H5) only in one (4%), both A(H5) and A(H9) in 13 (52%), and one A (nonsubtypeable) (4%). None of the participants were seropositive for AIVs.

**Conclusions:**

LBM workers had extensive exposure to AIVs, but none had serologic evidence of infection with A(H5N1) or A(H9N2) viruses circulating among poultry in Bangladesh. Ongoing surveillance of AIVs in LBMs and poultry workers is needed.

## 1 Introduction

Bangladesh is largely dependent on low‐cost protein consumption from live poultry production, and distribution, especially in urban settings. Avian influenza A viruses (AIVs) are endemic among poultry in Bangladesh [1], but only eight A(H5N1) and three A(H9N2) human infections were reported from Bangladesh up to 2024, including one fatal A(H5N1) case [2, 3]. However, in 2025, four A(H5N1) human cases in Bangladesh, all in children, were reported to WHO [4]. To date, all human cases of A(H5N1) and A(H9N2) in Bangladesh had a history of recent poultry exposure and four were live bird market (LBM) workers. LBMs have been identified as a potential source for persistence and transmission of A(H5N1) virus [5, 6]. Multiple subtypes of low pathogenic avian influenza (LPAI) and highly pathogenic avian influenza (HPAI) viruses have been detected in LBMs of Bangladesh [7]. In 2016, a parallel study of LBMs in Dhaka and Chattogram reported a high prevalence of both A(H5) and A(H9) viruses in poultry (63.2% pools positive for H9, 21.6% positive for H5, and 12.3% pools positive for both subtypes) and environmental samples (25% pools positive for H9 and 10.8% for H5) [8].

LBM workers are highly exposed to AIVs and have a higher risk of zoonotic spill over [9]. In an investigation of unusual crow deaths near LBMs in Dhaka, Bangladesh during January–February 2017, crows were observed feeding on poultry offal. Assessment of occupational risk identified 13.9% of LBM workers with avian influenza A virus detected by RT‐PCR in upper respiratory swabs (nasal/throat/both) [10]. Whether this indicated detection of infection or transient contamination was uncertain. Viable avian influenza A viruses were documented in air samples (*n* = 9/10, 90%) as well as in the environment where live poultry were sold [10], indicating potential for exposure to infectious AIVs and infection of workers. Some activities like slaughtering, defeathering, and eviscerating poultry were identified as having a high risk of exposure to AIVs [11]. An earlier study during 2012–2013 conducted at nine LBMs in Dhaka reported that 13% of the LBM workers with influenza‐like illness (ILI) had influenza A viral RNA detected in upper respiratory specimens [12]. However, as the presence of viral RNA in upper respiratory specimens of asymptomatic workers does not necessarily indicate infection, serological testing can help inform whether infection occurred [12].

A study by Papenburg et al. noted that microneutralization (MN) assays demonstrated a higher level of concordance with RT‐PCR and greater sensitivity in the serological diagnosis of A(H1N1)pdm09 virus infection compared to hemagglutination inhibition (HAI) assays [13]. Another study reported that neutralizing antibody titers were higher than HAI antibodies, also suggesting that MN assays may be more sensitive than HAI assays, particularly with low‐titered serum [14]. Nasreen et al. reported a cohort study of 404 LBM workers during 2009–2010 in Dhaka with 9 (2%) who were seropositive for A(H5N1) at baseline. Among 284 workers who completed the study and were seronegative at baseline, 6 (2%) had evidence of seroconversion [15].

We assessed virus detection by RT‐PCR in respiratory specimens and serological evidence of past avian influenza A virus infection by MN assay among an occupational population‐based sample of LBM workers in Dhaka, Bangladesh. We also collected follow‐up sera in those with influenza A RT‐PCR–positive respiratory specimens to determine whether serologic evidence of infection was present.

## 2 Methods

A cross‐sectional survey was conducted in 702 randomly selected LBM workers recruited from 42 LBMs in Dhaka City Corporation (DCC) from January to May 2017. The sample size for the virologic study was calculated based on an earlier study by Sturm‐Ramirez et al. during 2012–2013 in Dhaka [16], in which 4.5% of asymptomatic LBM workers had detectable AIV viral RNA in upper respiratory specimens. Assuming a minimum prevalence of A(H5N1) virus of 4.5% irrespective of symptoms, with 95% confidence, 2% precision, and 80% power, with a design effect of 1.5 and response rate of 85%, the sample size was estimated as 729. The LBMs were selected using probability proportional to the number of stalls in markets. We used a sampling frame of 106 LBMs in DCC having at least seven stalls available from the Sink Surveillance programmer of the Food and Agriculture Organization (FAO) in Bangladesh [8, 17]. Forty‐two LBMs were selected using probability proportional to size (PPS). A census of workers in each sampled LBM was taken from which 15 were selected through simple random sampling (five LBMs had two lots of 15 each selected by PPS of the size of the market). Respiratory specimens (nasal and throat swabs) were collected from each of the participants at recruitment. The sample size for the AIV seroprevalence study was estimated as 707 based on the earlier study conducted in 2009–2010 that reported an overall seroprevalence of A(H5N1) virus antibody among LBM worker of 5% [15], with a confidence level of 95%, a precision of 2.2%, and a design effect of 1.5. Blood specimens were collected from 695 workers (99% of all respondents in the virologic study) and follow‐up specimens were collected several weeks later from subjects who had at least one respiratory specimen positive for influenza A virus.

### 2.1 Air Sampling

Air samples were collected via cyclonic (liquid cyclone collector, CC, Midwest Micro‐tek, Brookings, South Dakota, USA, which processes air with a flow of 200 L/min) and impactor samplers (QuickTake30 Sample Pump, SKC Inc., with a 0.3‐μm pore size polytetrafluoroethylene [PTFE] filter preloaded in a 37‐mm testing cassette, which operates at an air flow of 20 L/min). Air samples were taken in each market on the same day workers were recruited. We used one cyclonic air sampler at 12 LBMs. For 30 LBMs, we used both cyclonic and impactor air samplers (Table S1a). Air samplers were placed 1.3 m above ground, approximately at the nose and mouth level of the LBM workers and 0.5–1.5 m from the poultry cages or baskets, adapted from a study in China [18]. The impactor air sampler was operated for 3 hours and the cyclonic air sampler for 30 min as recommended by the manufacturers. The five larger LBMs that had two lots of LBM workers selected by PPS had air samples collected on the same day each lot was recruited. Vaneometers were used with a moving replaceable vane (Vaneometer 480 Swing Vane Anemometer, Dwyer Instruments Inc) to record air velocity every 10 min for the cyclonic air sampler and every 30 min for the impactor air sampler. The vaneometers were placed at market entry and exit points and at the points where air samplers were placed. We recorded temperature and humidity at the beginning and end of the collection period using a temperature/humidity switch (°C, 230 Volts of Alternating Current).

### 2.2 Virological Testing of Air and Respiratory Samples

Air samples were tested with real‐time reverse transcription polymerase chain reaction (RT‐PCR) assay for influenza A viral RNA using Qiagen one step RT‐PCR kit (Qiagen, USA) at the National Reference Laboratory for Avian Influenza (WOAH Reference Laboratory), Bangladesh Livestock Research Institute (BLRI), Savar, Dhaka. The samples were screened first for the presence of the M gene by RT‐PCR assay using reference primers and probes. Then M gene positive samples were further assessed for A(H5), A(H7), and A(H9) subtypes by RT‐qPCR assay using specific primers and probes (See Table S7 for a list of primers and probes). For samples that were influenza A matrix (M) gene positive with a cycle threshold (Ct) value < 38, subtyping results were considered positive for A(H5) if the Ct value was < 38 and positive for A(H9) or A(H7) for Ct values < 40 [19].

All respiratory specimens were tested for influenza A and B viruses using RT‐PCR, with appropriate positive and negative controls and precautions to exclude contamination, at the One Health Laboratory of the Institute of Epidemiology, Disease Control and Research (IEDCR), Dhaka. Subtyping of M‐gene positive influenza A viruses was performed for A(H3), A(H1N1)pdm09, A(H5), A(H7), and A(H9), following the US CDC protocol and WHO guideline [20]. Reaction mixtures (25 μL) were prepared using the Ag‐Path‐ID One‐Step RT‐PCR Kit (Ambion, USA) and contained final concentrations: 12.5 μL of 2× RT‐PCR Buffer, 0.25 μL of 40‐μM reverse primer, 0.25 μL of 40‐μM forward primers, 0.25 μL of 10‐μM specific probe, 1 μL of 25× RT–PCR enzyme (Taq DNA polymerase and reverse transcriptase), and 5 μL of nucleic acid. Amplification was performed on ABI7500 Fast Real‐time PCR System (Applied Biosystems, USA) with the following cycling conditions: 40°C for 10 min, 95°C for 10 min, and 95°C for 15 s, followed by 55°C for 60 s (45 cycles). The positive controls for each virus were made of RNA extracts from noninfectious material for use with the CDC RT‐PCR method for detection and characterization of seasonal influenza viruses. Water was used as a negative control. Specimens were defined as influenza A(H5/H7/H9) positive with a Ct value < 38 using a subtype‐specific primer and A (nonsubtypeable) if influenza A M gene positive with Ct value < 38, but negative for A(H5/H7/H9/H1/H3) [21]. For the risk factor analysis we classified specimens that were positive for seasonal influenza A(H1), A(H1)pdm09, or A(H3) as negative for avian influenza A viruses (see Table S8 for list of primers and probes).

### 2.3 Serological Testing

All serum samples were tested by MN assay for neutralizing antibodies to A(H5N1) and A(H9N2) viruses at the WHO H5 Reference Laboratory, University of Hong Kong. A(H5N1) virus (A/crow/Bangladesh/689/16 H5N1, clade 2.3.2.1a) [22] and A(H9N2) virus (A/ck/Bangladesh/8222/17 H9N2, G1‐Western lineage) were isolated from a parallel poultry study in LBMs [23].

Influenza A(H5N1) virus stock was grown in Madin Darby Canine Kidney (MDCK) cells and titrated in quadruplicate in 96‐well microtiter plates in MDCK cells using serial 0.5 Log10 dilutions from 0.5 log to 8 log. Plates were observed in an inverted microscope for cytopathic effect (CPE) daily for 3 days. The highest dilution resulting in CPE in ≥ 50% of the inoculated wells was estimated using the Reed Muench method [24] and designated as one TCID_50_. Serial two‐fold dilutions of heat‐inactivated sera were made from 1:10 to 1:1280. The serum dilutions were mixed with an equal volume of 200 TCID_50_ influenza A(H5) virus. The serum virus mixture was incubated at 37°C for 1 h and then 35 μL of the mixture were added in quadruplicate to MDCK cell monolayers in 96‐well microtiter plates. After 1‐h adsorption, all medium was removed from the plates. One hundred microliters of culture medium was added to all wells and the plates were incubated for 3 days at 37°C in 5% CO_2_ in a humidified incubator. A virus back‐titration was carried out with culture medium in place of serum to confirm the input virus dose. The highest serum dilution protecting the cells from CPE forming in at least half of the wells at 3 days after infection was defined as the neutralizing antibody titer. Experiments involving highly pathogenic avian influenza A viruses were performed in a biosafety level 3 facility. A seropositive result was defined as a neutralizing antibody titer ≥ 1:40 [25]. The same procedures were followed for influenza A(H9N2) virus.

### 2.4 Ethical Aspects

Ethical approvals were obtained from the Institute of Epidemiology, Disease Control and Research (IEDCR), Bangladesh (Reference: IEDCR/IRB/2016/08); the Royal Veterinary College (RVC), UK (Reference: URN 2017 1691‐3), and the London School of Hygiene and Tropical Medicine (LSHTM), UK (LSHTM Ethics Ref: 14466). Only adult participants providing informed written consent were enrolled. If a worker aged < 18 years was selected, they were only enrolled if consent from a legal guardian was obtained.

### 2.5 Statistical Analysis

The baseline characteristics of the study population, and potential associations between market, market stall, and individual‐level factors and positive RT‐PCR results for A(H5), A(H9), or A (nonsubtypeable) were described. Associations between risk factors and MN assay results were also assessed. Odds ratios (ORs) (and 95% CI) were computed using random effects logistic regression, adjusting a priori for market‐level clustering using a random intercept, and age at baseline (correlated with duration of experience) [11] in order to examine market‐level factors. To assess stall and individual‐level factors, we adjusted for the following market‐level factors: presence of ducks (based on observation), market type (wholesale and retail or retail only), and size of markets (< 15 or 15 or more stalls per market) as a priori confounders.

We explored seroconversion rates by cross‐tabulating RT‐PCR results against baseline and follow‐up A(H5N1) and A(H9N2) serology results. All analyses were conducted using Stata 16.1 (College Station, Texas, USA).

## 3 Results

Among 42 LBMs, six reported market cleaning more than once daily, but only one used disinfectant (e.g., bleach), while others used only water. Three LBMs reported disposal of solid waste at least twice daily. Half of the markets had open drains. About 60% of stalls experienced poultry deaths in the week before the investigations.

The 42 LBMs had a median of 18 stalls (IQR = 11–25) per market, and 702 LBM workers were enrolled among a total of 2148 workers (97% response rate of those selected). Over half were recruited during the winter season (January–March), the rest during April and May 2017 (summer season). Most (70.7%, *n* = 496) participants worked in a retail market, the rest at mixed wholesale and retail markets. Nearly all were male (99.3%, *n* = 697) with a median age of 28 years (IQR = 22–38 years) and had worked in LBMs for a median of 8 years (IQR = 3–15 years). Only around 10% (*n* = 67) worked in stalls that sold and/or slaughtered ducks, but almost all participants were involved with poultry care, slaughtering and processing, and cleaning related activities (Tables 1, 2, Table S1b).

**TABLE 1:** Association between characteristics of live bird markets from a cross‐sectional survey of 702 randomly sampled live bird market workers in 42 markets in Dhaka in 2017 and workers with respiratory specimens positive for A(H5), A(H9), or A (nonsubtypeable) by RT‐PCR.^a^

	All	RT‐PCR result	OR^b^ (95% CI)	*p*
		Number of positive (%)		
**All**	702	99 (14.1)		
**Season**				
Winter (Jan–March)	400	61 (15.3)	1.75 (0.59–5.17)	
Summer (April–May)	302	38 (12.6)	1.00	0.310
**Selling ducks**				
Yes	493	92 (18.7)	7.33 (2.36–22.73)	0.001
No	209	7 (3.4)	1.00	
**Type of LBM**				
Wholesale & retail	206	37 (18.0)	2.15 (0.69–6.66)	0.186
Retail	496	62 (12.5)	1.00	
**Market size**				
More than 15 stalls	395	75 (19.0)	4.05 (1.47–11.14)	0.007
Up to 15 stalls	307	24 (7.8)	1.00	
**Use of disinfectant at LBM**				
Use only water or detergent	579	77 (13.3)	0.56 (0.16–1.99)	0.366
Use disinfectant	123	22 (17.9)	1.00	
**Selling geese**				
Yes	245	49 (20.0)	2.73 (0.97–1.03)	0.057
No	457	50 (10.9)	1.00	
**Selling nonpoultry birds** ^c^				
Yes	500	89 (17.8)	3.85 (1.30–11.34)	0.015
No	202	10 (5.0)	1.00	
**Presence of dead birds on market ground (observation)** ^d^				
Yes	80	26 (32.5)	5.70 (1.42–22.92)	0.014
No	622	73 (11.7)	1.00	
**Air sample PCR result for M gene**				
Positive	433	88 (20.3)	6.38 (2.48–16.44)	< 0.001
Negative	269	11 (4.1)	1.00	

^a^ Those with seasonal influenza positive RT‐PCR tests were excluded from the numerator.

^b^ ORs based on random‐effect modeling to account for market‐level clustering and adjusted for age.

^c^ Nonpoultry birds include quail and pigeons.

^d^ Research team observations during the survey period.

**TABLE 2:** Association between characteristics of the stalls where recruited personnel worked from a cross‐sectional survey of 702 randomly sampled live bird market workers in 42 markets in Dhaka in 2017 and workers with respiratory specimens positive for A(H5), A(H9), or A (nonsubtypeable) by RT‐PCR.^a^

		All	RT‐PCR number of positive (%)	OR^b^ (95% CI)	*p*	aOR^c^ (95% CI)	*p* ^d^
**All**		702	99 (14.1)				
**Stalls sell duck/geese**							
	Yes	74	16 (21.6)	1.33 (0.67–2.66)	0.414	1.18 (0.60–2.31)	0.635
	No	628	56 (8.9)	1.00		1.00	
**Defeathering machine with cover (observed)** Whether use defeathering machine in the stall and covered during defeathering process							
	Used defeathering machine and cover it	257	26 (10.1)	0.61 (0.35–1.06)	0.080	0.55 (0.32–0.96)	0.035
	Used defeathering machine but did not cover it	32	4 (12.5)	1.08 (0.27–4.30)		0.84 (0.25–2.86)	
	Did not use defeathering machine	413	69 (16.7)	1.00		1.00	
**Poultry boiled before defeathering**							
	Yes	322	33 (10.3)	0.61 (0.36–1.04)	0.072	0.57 (0.33–0.96)	0.035
	No	380	66 (17.4)	1.00		1.00	
**Are there separate slaughtering and selling areas in the stall (*n* missing = 1)**							
	Yes	430	54 (12.6)	0.69 (0.43–1.10)	0.118	0.71 (0.44–1.14)	0.153
	No	271	45 (16.6)	1.00		1.00	
**Birds removed during cleaning of poultry cages/areas (*n* missing = 7)**							
	Yes	291	34 (11.7)	0.86 (0.52–1.43)	0.1559	0.89 (0.53–1.47)	0.640
	No	404	63 (15.6)	1.00		1.00	

^a^ Those with seasonal influenza A positive RT‐PCR results were excluded from the numerator. None were positive for influenza B or influenza A(H7).

^b^ ORs based on random‐effect modeling to account for market‐level clustering and adjusted for age.

^c^ Random effects ORs adjusted for age, presence of duck in the LBM, type, and size of the LBM.

^d^ Test for trend if ordered categorical variable.

Among 42 LBMs, 25 (59.5%) were RT‐PCR positive for influenza A virus in one or more air samples, with 10 LBMs positive for A(H9) only (40%), one A(H5) only (4%), 13 for both A(H5) and A(H9) (52%), and one LBM had an A (nonsubtypeable) virus (4%). None were positive for influenza A(H7) virus (Table S1a). No clear seasonal patterns were seen (Table S1b).

Among the 702 enrolled workers, 204 (29.1%) reported any respiratory symptoms in the previous 10 days (runny nose: 152 [21.7%]; cough: 121 [17.2%]; sore throat: 54 [7.7%]; shortness of breath/dyspnea: 18 [2.6%]). The proportion with such symptoms was similar in those with respiratory specimens that tested RT‐PCR positive for influenza A virus (31.3%, 31/99) (excluding 4 people positive for seasonal influenza) and those with respiratory specimens that tested negative for influenza A virus (28.9%, 173/599). Among those who did not report any respiratory symptoms in the 10 days before respiratory specimen collection (*n* = 498/702, 70.9%), 13.7% (*n* = 68/498) had at least one respiratory specimen that was RT‐PCR positive for an avian influenza A virus (excluding those positive for seasonal influenza), compared with 15.2% (*n* = 31/204) (*p* = 0.711) who reported at least one respiratory symptom in the prior 10 days.

Among 702 participants, three had respiratory specimens that tested positive for A(H1) and one was positive for A(H3) (median Ct value: 31.75, IQR = 31.19–35.17*)*. Ninety‐nine (14.1%) were RT‐PCR positive for influenza A virus (H1 and H3 negative) in at least one respiratory specimen (nasal/throat/both). None were positive for influenza B. Of these 99, 28 (28.3%) were positive for A(H9) only, 2 (2%) were positive for A(H5) only, including 3 (3%) that were positive for both A(H5) and A(H9) (median Ct value: 35.05, IQR = 34.16–36.70*)*; 66 (66.7%) were positive for influenza A but were not subtypeable (median Ct value: 36.09, IQR = 34.89–36.75) and none were positive for influenza A(H7) (Table 2).

### 3.1 Association Between RT‐PCR–Positive (A[H5], A[H9], or A [Nonsubtypeable]) Respiratory Specimens and Poultry Handling

We examined factors related to the presence of any positive respiratory RT‐PCR result for A(H5), A(H9), or A (nonsubtypeable) in LBM workers. Participants with positive respiratory specimens after adjusting for age and clustering at the market level were more likely to work in LBMs where ducks (OR = 7.33, 95% CI = 2.36–22.73) or nonpoultry birds, including quail and pigeon (OR = 3.85, 95% CI = 1.30–11.34), were sold, in markets with > 15 stalls (OR = 4.05, 95% CI = 1.47–11.14), with observed dead birds (OR = 5.70, 95% CI = 1.42–22.92) and in markets with air samples that were influenza A virus positive by RT‐PCR (OR = 6.38, 95% CI = 2.48–16.44) (Table 1).

At the stall level after adjusting for market level factors including the presence of ducks, type, and size of markets, participants working in a stall, where a defeathering machine was used with a cover and poultry were boiled before defeathering, were less likely than those that did not have respiratory specimens positive for A(H5), A(H9), or A (nonsubtypeable) (aOR = 0.55, 95% CI = 0.32–0.96 and aOR = 0.57, 95% CI = 0.33–0.96) (Table 2).

At the individual level, there was insufficient evidence that high‐risk activities such as slaughtering, defeathering or eviscerating (aOR = 0.70, 95% CI = 0.29–1.67) were associated with testing positive for influenza A(H5), A(H9), or A (nonsubtypeable) by RT‐PCR in respiratory specimens (see Table S2 for further details).

### 3.2 Presence of A(H5N1) and A(H9N2) Virus Antibodies in LBM Workers

Blood specimens were obtained from 695 LBM participants in the cross‐sectional study, and none were seropositive for A(H5N1) or A(H9N2) by MN assay (27 [3.9%, 95% CI = 2.7–5.6%] had an A[H5N1] virus neutralizing antibody titer of 1:10, and the remainder had no detectable antibodies). No participants had detectable neutralizing antibodies to A(H9N2) virus (See Tables S3–5 for further details).

### 3.3 Follow‐Up Neutralizing Antibody Titers in Those With AIV Positive RT‐PCR Respiratory Specimens at Baseline

Follow‐up survey blood specimens were collected from 89 of the 103 (86.4%) participants with respiratory specimens that tested positive for influenza A virus. We excluded three participants (the fourth did not have a follow‐up serum specimen) with respiratory specimens positive for seasonal influenza A viruses during the baseline survey. Of the remaining 86 participants, blood specimens were collected after a median of 77 days (IQR = 56–175 days) from the initial survey, and none were seropositive for antibodies to A(H5N1) or A(H9N2) viruses. One worker had an A(H9) positive respiratory specimen with no detectable neutralizing antibodies at baseline and a neutralizing antibody titer of 1:10 to A(H5N1) at follow‐up; two had A (nonsubtypeable) virus detected in respiratory specimens and a titer of 1:10 to A(H5N1) virus at baseline and follow‐up (Table S5).

## 4 Discussion

We conducted a cross‐sectional survey among LBM workers in Bangladesh in 2017 when highly pathogenic avian influenza A(H5N1) virus, clade 2.3.2.1a, and low pathogenic avian influenza A(H9N2) virus, G1 “Western lineage,” were circulating among poultry [22, 26]. Air sampling findings and virological data indicated that LBM workers were highly exposed to AIVs but none had serological evidence of infection with A(H5N1) or A(H9N2) viruses at baseline or at follow‐up, in workers with detectable AIV viruses in respiratory specimens at baseline.

Among highly exposed LBM workers, having a respiratory specimen that tested positive for influenza A(H5), A(H9), or influenza A (nonsubtypeable) by RT‐PCR was significantly associated with market and stall level factors such as having air samples positive for A(H5) and A(H9) or influenza A (nonsubtypeable) viral RNA, selling ducks, selling nonpoultry birds, the presence of dead birds, and the larger size of the market (more than 15 stalls). Those who worked in stalls where poultry were boiled before defeathering or used a covered defeathering machine compared to no defeathering machine were significantly less likely to have positive RT‐PCR respiratory specimens. The association of RT‐PCR–positive human respiratory specimens with high‐risk poultry processing was not surprising in our study population because very limited prevention measures were used routinely as described by several earlier studies [10, 11, 27].

Results from a 2009 sero‐survey study from Bangladesh reported LBM workers with higher geometric mean titers of A(H5) neutralizing antibodies compared to farm poultry workers, although no poultry workers met the WHO criteria for a seropositive result (neutralizing antibody titer ≥ 1:40) [25, 28]. The same 2009 study reported that some of the market workers had a neutralizing antibody titer of ≥ 1:20, and the relative risk of having a neutralizing antibody titer to A(H5N1) virus of ≥ 1:20 among the market workers was higher among workers who reported that they slaughtered, defeathered or eviscerated poultry compared to workers who did not conduct those activities in the markets. A 2013 cohort study of LBM workers in Bangladesh reported that of 28 workers ascertained as having an acute influenza‐like illness, 3.5% (1/28) had detectable neutralizing antibody and HAI titers (both ≥ 1:40) for A(H5N1) and 42% (12/28) for A(H9N2) at baseline, but there was no evidence of antibody rise in convalescent sera collected 3–4 weeks later [12].

It is difficult to interpret low but detectable levels of influenza A(H5N1) antibodies below the threshold for a seropositive result especially in cross‐sectional studies. Detection of low levels of A(H5N1) neutralizing antibodies could reflect declining titers from past influenza A(H5N1) virus infection or be due to a mild antibody titer rise from asymptomatic influenza A(H5) virus infection or mild clinical illness [29]. Low antibody titers could also be due to cross‐reactive antibodies from previous infection with seasonal influenza A viruses or nonspecific cross‐reactivity and not represent past exposure or infection with influenza A(H5N1) viruses [20].

Our seroprevalence results are consistent with a meta‐analysis of seroprevalence studies indicating that serological evidence of A(H5N1) virus infections prior to the emergence of clade 2.3.4.4b viruses is rare [30]. From 1997 to 2020, several studies reported zero to low influenza A(H5N1) antibody seroprevalence, and other studies thereafter reported detectable but low seroprevalence of possible asymptomatic infections in LBM workers that varied by clade, being higher in those exposed to clade 0 viruses such as during the Hong Kong influenza A(H5N1) outbreak in 1997 [30, 31, 32]. Since 2022, global dissemination of highly pathogenic avian influenza (HPAI) A(H5N1) virus, clade 2.3.4.4b, has been documented among wild birds, and poultry, spillover to a wide range of mammals, and between 2022 and 2023, a seroprevalence of 4.6% was noted against this clade in Egyptian LBM workers (*N* = 394) based on neutralizing antibody titers of ≥ 1:40, including 13/164 (8%) with evidence of seroconversion [31].

We found that a higher percentage of workers' respiratory specimens (3.8%) were positive for influenza A(H9) virus than A(H5) virus. The findings were consistent with a parallel study of poultry and the surrounding environment from February to March 2016 that reported a higher prevalence of influenza A(H9) than A(H5) viruses in selected LBMs of Dhaka and Chattogram [8]. Studies have reported the emergence of influenza A(H9N2) viruses with an enhanced receptor binding preference for human‐like receptors [33] with the capacity to bind to both α,2‐3 linked sialic acid (avian‐like) and α,2‐6 sialic acid linked receptors (most prevalent in the human upper respiratory tract) [34] although no neutralizing antibodies to influenza A(H9N2) virus were detected indicating asymptomatic or mild infections with influenza A(H9N2) virus [35].

Our study has a number of limitations. Air sampling was carried out in markets from January to May 2017. Although patterns in the main viruses detected may have varied by season (winter vs. summer), we were unable to assess this. There was variation in the number of air samples taken with one sampler only available during some of the field work. Testing of the five larger markets was carried out twice as we sampled two lots of individuals at different times in each market based on sampling probability proportion to market size.

In our study, recruitment of workers present at LBMs on the day of the survey might have failed to identify any potentially infected person if they were absent from work due to illness. We identified a high proportion of influenza A positive respiratory specimens that were nonsubtypeable by RT‐PCR. We hypothesized that (a) the low levels of influenza A viral RNA that yielded high Ct values could not be subtyped for influenza A(H5), A(H7), and A(H9) or (b) that it might be possible that other influenza A virus subtypes (AIVs) were present that were not tested for—such as A(H4), A(H6), A(H10), A(H11), etc. [7, 36].

While the WHO recommends the use of MN assay plus a different confirmatory serologic assay for detection of A(H5N1) in a single serum specimen, we only used the MN assay. Because of resource limitations, we collected follow‐up serum specimens only from those who were initially positive for M‐gene with RT‐PCR with respiratory specimens (irrespective of the presence or absence of symptoms). For serological confirmation of a symptomatic individual with suspected A(H5N1) virus infection, WHO recommends paired sera collection, a ≥ 4‐fold rise in neutralizing antibody titers (or equivalent) to an influenza A(H5) virus antigenically similar to the virus the person was exposed to, with a convalescent neutralizing titer ≥ 1:40 [25]. An acute serum specimen should be collected within 7 days of symptom onset and a convalescent serum specimen collected ideally 21–28 days after symptom onset [25]. The delay in follow‐up serum collection in our study might have led to missed evidence of seroconversion. Antibody responses wane faster in those individuals who are mildly symptomatic or asymptomatic compared to those with severe illness [29]. In addition, although transportation of sera from Bangladesh to the University of Hong Kong serology laboratory was performed using a standard protocol, there may have been specimen degradation during transportation or during the long storage time, as the sera collected in 2017 were tested in 2021. Because of resource limitations, we were not able to perform the MN assay against seasonal influenza A(H1) and A(H3) viruses. Therefore, we cannot exclude the possibility that the detection of A(H5N1) virus neutralizing antibody titers of 1:10 might have been due to cross‐reactivity from antibodies to seasonal influenza A viruses [20, 37]. Including an unexposed control group might help exclude cross‐reactivity caused by infection with other influenza viruses or influenza vaccination [38]. Our study did not have such a control group.

## 5 Conclusions

In summary, we detected evidence of extensive occupational exposures to avian influenza A(H5N1) and A(H9N2) viruses through air sampling and upper respiratory tract sampling among LBM workers in Bangladesh during 2017. However, no serologic evidence of influenza A(H5N1) or A(H9N2) virus infection was identified by MN assays for the influenza A(H9N2) and A(H5N1) viruses circulating among poultry in LBMs in Bangladesh at that time. Further virologic and serologic studies are needed to assess the potential for human infections in exposed poultry workers and as avian influenza A viruses evolve.

## Author Contributions

**Mahbubur Rahman:** conceptualization, methodology, data curation, investigation, formal analysis, writing – original draft, software, project administration, supervision, project administration, funding acquisition, writing – review and editing. **Timothy M. Uyeki:** formal analysis, writing – review and editing, methodology, validation. **Malik Peiris:** methodology, formal analysis, writing – review and editing, validation. **Min Kim:** formal analysis, writing – review and editing. **Sudipta Sarkar:** investigation, writing – review and editing. **Md Giasuddin:** investigation, writing – review and editing. **Md Ahasanul Hoque:** supervision, funding acquisition, resources, writing – review and editing. **Montse Torremorell:** resources, writing – review and editing. **Mahmudur Rahman:** conceptualization, methodology, data curation, investigation, formal analysis, writing – original draft, software, project administration, supervision, project administration, funding acquisition, writing – review and editing. **Guillaume Fournié:** funding acquisition, project administration, resources, writing – review and editing. **Meerjady Sabrina Flora:** project administration, supervision, writing – review and editing. **Punam Mangtani:** conceptualization, methodology, supervision, funding acquisition, resources, formal analysis, writing – review and editing, project administration.

## Consent

All participants provided informed written consent prior to their inclusion in the study.

## Conflicts of Interest

The authors declare no conflicts of interest.

## Peer Review

The peer review history for this article is available at https://www.webofscience.com/api/gateway/wos/peer‐review/10.1111/irv.70189.

## Supporting information


**Supplementary Table 1a**: Air samplers used, and number of air samples collected from 42 LBMs.
**Supplementary Table 1b**: Monthly distribution of LBM air sampling results for influenza A(H5), influenza A(H9), and influenza A (nonsubtypeable)*.
**Supplementary Table 2**: Association between individual characteristics of participants recruited from a cross‐sectional survey of 702 randomly sampled live bird market workers in 42 markets in Dhaka and workers with respiratory specimens positive for A(H5), A(H9), or A (nonsubtypeable) by RT‐PCR*.
**Supplementary Table 3**: Live bird market level characteristics of workers with a neutralizing antibody titer to influenza A(H5N1) virus of 1:10 by microneutralization assay (*N* = 695).
**Supplementary Table 4**: Stall level characteristics of workers with a neutralizing antibody titer to influenza A(H5N1) virus of 1:10 by microneutralization assay (*N* = 695).
**Supplementary Table 5**: Individual level characteristics of workers with a neutralizing antibody titer to influenza A(H5N1) virus of 1:10 by microneutralization assay (*N* = 695).
**Supplementary Table 6**: Influenza A(H5N1) neutralizing antibody titers at baseline and follow‐up in LBM workers with respiratory specimens positive for A(H5), A(H9), or A (nonsubtypeable) by RT‐PCR at baseline (*N* = 86).
**Supplementary Table 7**: Primers and probes for RT‐qPCR test for air samples.
**Supplementary Table 8**: Primers and probes for RT‐PCR test for human respiratory samples.

## Data Availability

Data supporting the conclusions of this article are available on reasonable request. To protect patients' privacy, the ethical approval obtained for this study prevents human data from being disclosed publicly.

## References

[1] R. Parvin , M. Nooruzzaman , C. K. Kabiraj , et al., “Controlling Avian Influenza Virus in Bangladesh: Challenges and Recommendations,” Viruses 12, no. 7 (2020): 751. 32664683 10.3390/v12070751 PMC7412482

[2] A. Chakraborty , M. Rahman , M. J. Hossain , et al., “Mild Respiratory Illness Among Young Children Caused by Highly Pathogenic Avian Influenza A (H5N1) Virus Infection in Dhaka, Bangladesh, 2011,” Journal of Infectious Diseases 216, no. suppl_4 (2017): S520–S528. 28934459 10.1093/infdis/jix019 PMC11753186

[3] World Health Organization , Cumulative Number of Confirmed Human Cases for Avian Influenza A(H5N1) Reported to WHO, 2003‐2025, 25 August 2025, accessed September 10, 2025, (WHO, 2025), https://www.who.int/publications/m/item/cumulative‐number‐of‐confirmed‐human‐cases‐for‐avian‐influenza‐a(h5n1)‐reported‐to‐who‐‐2003‐2025‐‐25‐august‐2025.

[4] Centers for Disease Control and Prevention . Global Human Cases With Influenza A(H5N1), 1997–2025. Updated September 22, 2025, accessed September 30, 2025, (Centers for Disease Control and Prevention, 2025), https://www.cdc.gov/bird‐flu/php/surveillance/chart‐epi‐curve‐ah5n1.html.

[5] P. K. Biswas , J. P. Christensen , S. S. Ahmed , et al., “Avian Influenza Outbreaks in Chickens, Bangladesh,” Emerging Infectious Diseases 14, no. 12 (2008): 1909–1912. 19046518 10.3201/eid1412.071567 PMC2634614

[6] G. Fournié , J. Guitian , S. Desvaux , et al., “Identifying Live Bird Markets With the Potential to Act as Reservoirs of Avian Influenza A (H5N1) Virus: A Survey in Northern Viet Nam and Cambodia,” PLoS ONE 7, no. 6 (2012): e37986. 22675502 10.1371/journal.pone.0037986 PMC3366999

[7] N. A. Gerloff , S. U. Khan , N. Zanders , et al., “Genetically Diverse Low Pathogenicity Avian Influenza A Virus Subtypes Co‐Circulate Among Poultry in Bangladesh,” PLoS ONE 11, no. 3 (2016): e0152131. 27010791 10.1371/journal.pone.0152131 PMC4806916

[8] Y. Kim , P. K. Biswas , M. Giasuddin , et al., “Prevalence of Avian Influenza A(H5) and A(H9) Viruses in Live Bird Markets, Bangladesh,” Emerging Infectious Diseases 24, no. 12 (2018): 2309–2316. 30457545 10.3201/eid2412.180879 PMC6256373

[9] X. F. Wan , L. Dong , Y. Lan , et al., “Indications That Live Poultry Markets Are a Major Source of Human H5N1 Influenza Virus Infection in China,” Journal of Virology 85, no. 24 (2011): 13432–13438. 21976646 10.1128/JVI.05266-11 PMC3233185

[10] M. Rahman , P. Mangtani , T. M. Uyeki , et al., “Evaluation of Potential Risk of Transmission of Avian Influenza A Viruses at Live Bird Markets in Response to Unusual Crow Die‐Offs in Bangladesh,” Influenza and Other Respiratory Viruses 14, no. 3 (2020): 349–352. 31912608 10.1111/irv.12716 PMC7182606

[11] X. Zhou , Y. Wang , H. Liu , et al., “Effectiveness of Market‐Level Biosecurity at Reducing Exposure of Poultry and Humans to Avian Influenza: A Systematic Review and Meta‐Analysis,” Journal of Infectious Diseases 218, no. 12 (2018): 1861–1875. 29986030 10.1093/infdis/jiy400

[12] M. Z. Hassan , K. Sturm‐Ramirez , M. S. Islam , et al., “Interpretation of Molecular Detection of Avian Influenza A Virus in Respiratory Specimens Collected From Live Bird Market Workers in Dhaka, Bangladesh: Infection or Contamination?,” International Journal of Infectious Diseases: IJID: Official Publication of the International Society for Infectious Diseases 136 (2023): 22–28. 37652093 10.1016/j.ijid.2023.08.020 PMC11849796

[13] J. Papenburg , M. Baz , M. E. Hamelin , et al., “Evaluation of Serological Diagnostic Methods for the 2009 Pandemic Influenza A (H1N1) Virus,” Clinical and Vaccine Immunology 18, no. 3 (2011): 520–522. 21228145 10.1128/CVI.00449-10 PMC3067392

[14] I. Stephenson , A. Heath , D. Major , et al., “Reproducibility of Serologic Assays for Influenza Virus A (H5N1),” Emerging Infectious Diseases 15, no. 8 (2009): 1252–1259. 19751587 10.3201/eid1508.081754 PMC2815968

[15] S. Nasreen , S. U. Khan , S. P. Luby , et al., “Highly Pathogenic Avian Influenza A(H5N1) Virus Infection Among Workers at Live Bird Markets, Bangladesh, 2009‐2010,” Emerging Infectious Diseases 21, no. 4 (2015): 629–637. 25811942 10.3201/eid2104.141281 PMC4378465

[16] K. M. Sturm‐Ramirez , S. Afreen , M. Z. Rahman , et al., “Avian Influenza at the Animal‐Human Interface: Investigations Among Poultry Workers in Live Bird Markets in Dhaka City, Bangladesh, 2012‐13,” in Paper presented at: Options for the Control of Influenza; 5‐10 September, 2013; Cape Town, South Africa (International Society for Respiratory Viruses, 2013).

[17] M. Osmani , H. Akwar , Z. Hasan , S. Chakma , M. M. Hossain , and E. Brum , “Sink Surveillance, an Innovative Approach to Identify HPAI and Other Emerging Zoonotic Pathogens in Live Bird Markets in Bangladesh,” in Paper presented at: Prince Mahidol Award Conference (PMAC)29 January ‐ 3 February 2018; Bangkok, Thailand (Prince Mahidol Award Foundation, 2018).

[18] J. W. J. Zhou , X. Zeng , G. Huang , et al., “Isolation of H5N6, H7N9 and H9N2 Avian Influenza A Viruses From Air Sampled at Live Poultry Markets in China, 2014 and 2015,” Eurosurveillance 21, no. 35 (2016): pii=30331. 10.2807/1560-7917.ES.2016.21.35.30331 PMC5015459 27608369

[19] I. Monne , S. Ormelli , A. Salviato , et al., “Development and Validation of a One‐Step Real‐Time PCR Assay for Simultaneous Detection of Subtype H5, H7, and H9 Avian Influenza Viruses,” Journal of Clinical Microbiology 46, no. 5 (2008): 1769–1773. 18367569 10.1128/JCM.02204-07 PMC2395090

[20] World Health Organization , Recommendations and Laboratory Procedures for Detection of Avian Influenza A(H5N1) Virus in Specimens From Suspected Human Cases (WHO, 2007).

[21] World Health Organization . WHO Information for Molecular Diagnosis of Influenza Virus ‐ Update. May 2015 (WHO, 2015).

[22] J.‐H. Kwon , D.‐H. Lee , M. F. Criado , et al., “Genetic Evolution and Transmission Dynamics of Clade 2.3.2.1a Highly Pathogenic Avian Influenza A/H5N1 Viruses in Bangladesh,” Virus Evolution 6, no. 2 (2020): veaa046. 34127940 10.1093/ve/veaa046 PMC7474931

[23] R. N. Ripa , J. E. Sealy , J. Raghwani , et al., “Molecular Epidemiology and Pathogenicity of H5N1 and H9N2 Avian Influenza Viruses in Clinically Affected Chickens on Farms in Bangladesh,” Emerging Microbes & Infections 10, no. 1 (2021): 2223–2234. 34753400 10.1080/22221751.2021.2004865 PMC8635652

[24] L. J. Reed and H. Muench , “A Simple Method of Estimating Fifty Per Cent Endpoints,” American Journal of Epidemiology 27, no. 3 (1938): 493–497.

[25] Global Influenza Programme , WHO Case Definition for Human Infections With Avian Influenza A(H5) Virus Requiring Notification Under IHR (World Health Organization, 2005), https://www.who.int/teams/global‐influenza‐programme/avian‐influenza/case‐definitions#:~:text=A%20person%20with%20a%20laboratory‐confirmed%20infection%20with%20an,serological%20testing%20of%20paired%20acute%20and%20convalescent%20serum. Published 2024. Updated 4 November 2024.

[26] T. H. P. Peacock , J. James , J. E. Sealy , and M. Iqbal , “A Global Perspective on H9N2 Avian Influenza Virus,” Viruses 11, no. 7 (2019): 620. 31284485 10.3390/v11070620 PMC6669617

[27] G. Fournie , E. Hog , T. Barnett , D. U. Pfeiffer , and P. Mangtani , “A Systematic Review and Meta‐Analysis of Practices Exposing Humans to Avian Influenza Viruses, Their Prevalence, and Rationale,” American Journal of Tropical Medicine and Hygiene 97, no. 2 (2017): 376–388. 28749769 10.4269/ajtmh.17-0014 PMC5544094

[28] S. Nasreen , S. Uddin Khan , E. Azziz‐Baumgartner , et al., “Seroprevalence of Antibodies Against Highly Pathogenic Avian Influenza A (H5N1) Virus Among Poultry Workers in Bangladesh, 2009,” PLoS ONE 8, no. 9 (2013): e73200. 24039887 10.1371/journal.pone.0073200 PMC3764173

[29] P. Buchy , S. Vong , S. Chu , et al., “Kinetics of Neutralizing Antibodies in Patients Naturally Infected by H5N1 Virus,” PLoS ONE 5, no. 5 (2010): e10864. 20532246 10.1371/journal.pone.0010864 PMC2879427

[30] X. Chen , W. Wang , Y. Wang , et al., “Serological Evidence of Human Infections With Highly Pathogenic Avian Influenza A(H5N1) Virus: A Systematic Review and Meta‐Analysis,” BMC Medicine 18, no. 1 (2020): 377. 33261599 10.1186/s12916-020-01836-y PMC7709391

[31] M. Gomaa , Y. Moatasim , A. El Taweel , et al., “We Are Underestimating, Again, the True Burden of H5N1 in Humans,” BMJ Global Health 8, no. 8 (2023): e013146. 10.1136/bmjgh-2023-013146 PMC10465887 37643809

[32] A. M. Mellis , J. Coyle , K. E. Marshall , et al., “Serologic Evidence of Recent Infection With Highly Pathogenic Avian Influenza A(H5) Virus Among Dairy Workers ‐ Michigan and Colorado, June‐August 2024,” MMWR. Morbidity and Mortality Weekly Report 73, no. 44 (2024): 1004–1009. 39509348 10.15585/mmwr.mm7344a3 PMC11542770

[33] X. Sun , J. A. Belser , and T. R. Maines , “Adaptation of H9N2 Influenza Viruses to Mammalian Hosts: A Review of Molecular Markers,” Viruses 12, no. 5 (2020): 541. 32423002 10.3390/v12050541 PMC7290818

[34] P. J. Blair , S. D. Putnam , W. S. Krueger , et al., “Evidence for Avian H9N2 Influenza Virus Infections Among Rural Villagers in Cambodia,” Journal of Infection and Public Health 6, no. 2 (2013): 69–79. 23537819 10.1016/j.jiph.2012.11.005 PMC3612269

[35] S. U. Khan , B. D. Anderson , G. L. Heil , S. Liang , and G. C. Gray , “A Systematic Review and Meta‐Analysis of the Seroprevalence of Influenza A(H9N2) Infection Among Humans,” Journal of Infectious Diseases 212, no. 4 (2015): 562–569. 25712969 10.1093/infdis/jiv109 PMC4598807

[36] R. Parvin , J. A. Begum , E. H. Chowdhury , M. R. Islam , M. Beer , and T. Harder , “Co‐Subsistence of Avian Influenza Virus Subtypes of Low and High Pathogenicity in Bangladesh: Challenges for Diagnosis, Risk Assessment and Control,” Scientific Reports 9, no. 1 (2019): 8306. 31165743 10.1038/s41598-019-44220-4 PMC6549172

[37] J. H. C. M. Kreijtz , R. A. M. Fouchier , and G. F. Rimmelzwaan , “Immune Responses to Influenza Virus Infection,” Virus Research 162, no. 1 (2011): 19–30. 21963677 10.1016/j.virusres.2011.09.022

[38] M. R. Gomaa , A. S. Kayed , M. A. Elabd , et al., “Avian Influenza A(H5N1) and A(H9N2) Seroprevalence and Risk Factors for Infection Among Egyptians: A Prospective, Controlled Seroepidemiological Study,” Journal of Infectious Diseases 211, no. 9 (2015): 1399–1407. 25355942 10.1093/infdis/jiu529 PMC4462653

