# Supplementary material for: A Cross‐Sectional Virological and Sero‐Epidemiological Study of Exposures to Avian Influenza A(H5N1) and A(H9N2) Viruses in Live Bird Market Workers in Dhaka, Bangladesh
Source: Influenza Other Respir Viruses. 2025 Nov 16;19(11):e70189. doi: 10.1111/irv.70189 (PMC12620130; doi:10.1111/irv.70189)
Supplement: Supplementary file 1 — Supplementary Table 1a: Air samplers used, and number of air samples collected from 42 LBMs. Supplementary Table 1b: Monthly distribution of LBM air sampling results for influenza A(H5), influenza A(H9), and influenza A (nonsubtypeable)*. Supplementary Table 2: Association between individual characteristics of participants recruited from a cross‐sectional survey of 702 randomly sampled live bird market workers in 42 markets in Dhaka and workers with respiratory specimens positive for A(H5), A(H9), or A (nonsubtypeable) by RT‐PCR*. Supplementary Table 3: Live bird market level characteristics of workers with a neutralizing antibody titer to influenza A(H5N1) virus of 1:10 by microneutralization assay (N = 695). Supplementary Table 4: Stall level characteristics of workers with a neutralizing antibody titer to influenza A(H5N1) virus of 1:10 by microneutralization assay (N = 695). Supplementary Table 5: Individual level characteristics of workers with a neutralizing antibody titer to influenza A(H5N1) virus of 1:10 by microneutralization assay (N = 695). Supplementary Table 6: Influenza A(H5N1) neutralizing antibody titers at baseline and follow‐up in LBM workers with respiratory specimens positive for A(H5), A(H9), or A (nonsubtypeable) by RT‐PCR at baseline (N = 86). Supplementary Table 7: Primers and probes for RT‐qPCR test for air samples. Supplementary Table 8: Primers and probes for RT‐PCR test for human respiratory samples. [file IRV-19-e70189-s001.docx]

**Title:** A cross-sectional virological and sero-epidemiological study of exposures to avian influenza A(H5N1) and A(H9N2) viruses in live bird market workers in Dhaka, Bangladesh

**Running Title**: Avian influenza A virus exposure in live bird market workers

**Authors:** Mahbubur Rahman, Timothy M. Uyeki, Malik Peiris, Jacqueline M Cardwell, Patrick Nguipdop-Djomo, Min Kim, A S M Alamgir, A K M Muraduzzaman, Sudipta Sarkar, Md Giasuddin, Md Ahasanul Hoque, Montse Torremorell, Mahmudur Rahman, Guillaume Fournié, Dirk U Pfeiffer, Meerjady Sabrina Flora, Punam Mangtani

**Supplementary Files**

**Air Sampling Results**

**Supplementary Table 1a:** Air samplers used, and number of air samples collected from 42 LBMs.

| **Number of air samples** | | **Total number of samples per LBM** | **Number of LBMs** |
| --- | --- | --- | --- |
| **Cyclonic** | **Impactor** |  |  |
| 1 |  | 1 | 10 |
| 2 |  | 2 | 2 |
| 1 | 1 | 2 | 27 |
| 2 | 2 | 4 | 3 |

**Supplementary Table 1b: Monthly distribution of LBM air sampling results for influenza A(H5), influenza A(H9), and influenza A(non-subtypeable)***

| **Month** | **Negative** | **A(H5) positive** | **A(H9) positive** | **Both A(H5) & A(H9) positive** | **A positive**  **(Non- subtypeable)** | **Total** |
| --- | --- | --- | --- | --- | --- | --- |
| Jan-17 | 1 | 0 | 0 | 5 | 0 | **6** |
| Feb-17 | 2 | 1 | 0 | 6 | 1 | **10** |
| Mar-17 | 5 | 0 | 0 | 2 | 0 | **7** |
| Apr-17 | 2 | 0 | 8 | 0 | 0 | **10** |
| May-17 | 7 | 0 | 2 | 0 | 0 | **9** |
| **Total** | **17** | **1** | **10** | **13** | **1** | **42** |

*****None were positive for A(H7)

**Supplementary Table 2**: Association between individual characteristics of participants recruited from a cross-sectional survey of 702 randomly sampled live bird market workers in 42 markets in Dhaka and workers with respiratory specimens positive for A(H5), A(H9) or A(non-subtypeable) by RT-PCR*

|  | | | All | RT-PCR result Number positive (%) | OR^†^ (95% CI) | p-value | aOR^‡^ (95% CI) | p-value^§^ |
| --- | --- | --- | --- | --- | --- | --- | --- | --- |
| **All** | | | 702 | 99 (14.1) |  |  |  |  |
| **Age (years), median (IQR)** | | | 28 (22,38) | 30 (23,40) | 1.01 (0.99-1.03) | 0.480 | 1.01 (0.99-1.03) | 0.552 |
| **Current smoker** | | |  |  |  |  |  |  |
|  | Yes | | 309 | 37 (12.0) | 0.75 (0.46-1.20) | 0.229 | 0.73 (0.45-1.18) | 0.195 |
|  | No | | 393 | 62 (15.8) | 1.00 |  | 1.00 |  |
| **No of years worked in the market, median (IQR)** | | | 8 (3,15) | 10 (5,18) | 1.01 (0.98-1.04) | 0.598 | 1.00 (0.96-1.03) | 0.889 |
| **No of years worked in the market** | | |  |  |  |  |  |  |
| <10 years | | | 359 | 44 (12.3) | 1.00 |  | 1.00 |  |
| ≥10 years | | | 343 | 55 (16.0) | 1.18 (0.67-2.07) | 0.575 | 1.16 (0.66-2.04) | 0.618 |
| **Average working hours, mean (SD)** | | | 12.2 (2.5) | 12.3 (2.4) | 1.00 (0.90-1.10) | 0.932 | 0.98 (0.89-1.08) | 0.693 |
| **Average working hours** | | |  |  |  |  |  |  |
| Up to 8 hours | | | 64 | 7 (10.9) | 1.00 |  | 1.00 |  |
| >8 hours | | | 638 | 92 (14.4) | 1.31 (0.53-3.23) | 0.559 | 1.23 (0.50-3.02) | 0.650 |
| **Poultry care** | | |  |  |  |  |  |  |
|  | Yes | | 691 | 96 (13.9) | 0.30 (0.06-1.51) | 0.145 | 0.38 (0.08-1.80) | 0.221 |
|  | No | | 11 | 3 (27.3) | 1.00 |  | 1.00 |  |
|  | **Carrying poultry** | |  |  |  |  |  |  |
|  |  | Yes, with hands and/or baskets | 462 | 65 (14.1) | 0.88 (0.52-1.48) | 0.628 | 0.84 (0.50-1.42) | 0.518 |
|  |  | No | 240 | 34 (14.2) | 1.00 |  | 1.00 |  |
|  | **Feeding poultry** | |  |  |  |  |  |  |
|  |  | Yes | 626 | 83 (13.3) | 0.62 (0.32-1.22) | 0.166 | 0.63 (0.32-1.23) | 0.176 |
|  |  | No | 76 | 16 (21.1) | 1.00 |  | 1.00 |  |
|  | **Selling poultry** | |  |  |  |  |  |  |
|  |  | Yes | 673 | 95 (14.1) | 1.07 (0.32-3.59) | 0.908 | 1.10 (0.33-3.66) | 0.871 |
|  |  | No | 28 | 4 (14.3) | 1.00 |  | 1.00 |  |
| **Poultry slaughtering and processing** | | |  |  |  |  |  |  |
|  | Yes | | 657 | 89 (13.6) | 0.66 (0.27-1.60) | 0.359 | 0.70 (0.29-1.67) | 0.420 |
|  | No | | 45 | 10 (22.2) | 1.00 |  | 1.00 |  |
|  | **Slaughtering** | |  |  |  |  |  |  |
|  |  | Yes | 655 | 89 (13.6) | 0.69 (0.29-1.66) | 0.409 | 0.72 (0.30-1.72) | 0.466 |
|  |  | No | 47 | 10 (21.3) | 1.00 |  | 1.00 |  |
|  | **Scalding/boiling** | |  |  |  |  |  |  |
|  |  | Yes | 285 | 31 (10.9) | 0.71 (0.42-1.22) | 0.220 | 0.68 (0.40-1.17) | 0.165 |
|  |  | No | 417 | 68 (16.3) | 1.00 |  | 1.00 |  |
|  | **Defeathering (verbal report)** | |  |  |  |  |  |  |
|  |  | Yes, with only hands | 318 | 50 (15.7) | 1.09 (0.54-2.19) | 0.644 | 1.17 (0.58-2.33) | 0.475 |
|  |  | Yes, with machine (+ hands) | 275 | 32 (11.6) | 0.89 (0.42-1.91) |  | 0.88 (0.41-1.87) |  |
|  |  | No | 109 | 17 (15.6) | 1.00 |  | 1.00 |  |
|  | **Eviscerating** | |  |  |  |  |  |  |
|  |  | Yes | 590 | 81 (13.7) | 0.96 (0.50-1.88) | 0.916 | 1.01 (0.52-1.95) | 0.983 |
|  |  | No | 112 | 18 (16.1) | 1.00 |  | 1.00 |  |
|  | **Washing processed poultry** | |  |  |  |  |  |  |
|  |  | Yes | 348 | 51 (14.7) | 1.30 (0.80-2.14) | 0.291 | 1.25 (0.76-2.05) | 0.375 |
|  |  | No | 354 | 48 (13.6) | 1.00 |  | 1.00 |  |
| **Cleaning related activities** | | |  |  |  |  |  |  |
|  | Yes | | 627 | 82 (13.1) | 0.58 (0.30-1.15) | 0.121 | 0.58 (0.30-1.14) | 0.115 |
|  | No | | 75 | 17 (22.7) | 1.00 |  | 1.00 |  |
|  | **Cleaning feeding tray/water container** | |  |  |  |  |  |  |
|  |  | Yes | 569 | 73 (12.8) | 0.70 (0.40-1.24) | 0.222 | 0.72 (0.41-1.26) | 0.251 |
|  |  | No | 133 | 26 (19.6) | 1.00 |  | 1.00 |  |
|  | **Collecting/transporting carcasses and offal** | |  |  |  |  |  |  |
|  |  | Yes | 564 | 72 (12.8) | 0.63 (0.35-1.12) | 0.112 | 0.65 (0.37-1.15) | 0.141 |
|  |  | No | 138 | 27 (19.6) | 1.00 |  | 1.00 |  |
|  | **Collecting/transporting faces and feathers** | |  |  |  |  |  |  |
|  |  | Yes | 526 | 68 (12.9) | 0.71 (0.41-1.22) | 0.218 | 0.73 (0.43-1.25) | 0.255 |
|  |  | No | 176 | 31 (17.6) | 1.00 |  | 1.00 |  |
|  | **Cleaning poultry cages/baskets/keeping area** | |  |  |  |  |  |  |
|  |  | Yes | 542 | 72 (13.3) | 0.72 (0.41-1.27) | 0.257 | 0.74 (0.42-1.31) | 0.304 |
|  |  | No | 160 | 27 (16.9) | 1.00 |  |  |  |

^†^ORs were adjusted for age and market-level clustering using a random-effect.

^‡^Random effects ORs were adjusted for age, presence of duck in the LBM, type and size of the LBM.

^§^Test for trend if ordered categorical variable

*Those with seasonal influenza A positive RT-PCR results were excluded from the numerator. None were positive for influenza B or influenza A(H7).

**Serology results**

**Supplementary Table 3**: Live bird market level characteristics of workers with a neutralizing antibody titer to influenza A(H5N1) virus of 1:10 by microneutralization assay (N=695)

| Market level factors | | All | Number with a neutralizing antibody titer to influenza A(H5N1) virus of 1:10 (%) | OR^†^ (95% CI) | p-value |
| --- | --- | --- | --- | --- | --- |
|  |  |  |  |  |  |
| **All** | | 695 | 27 (3.9) |  |  |
| **Selling ducks** | |  |  |  |  |
|  | Yes | 492 | 18 (3.7) | 0.78 (0.23-2.58) | 0.678 |
|  | No | 203 | 9 (4.4) | 1.00 |  |
| **Type of LBM** | |  |  |  |  |
|  | Wholesale & retail | 206 | 9 (4.4) | 1.22 (0.34-4.41) | 0.765 |
|  | Retail | 489 | 18 (3.7) | 1.00 |  |
| **Market size** | |  |  |  |  |
|  | Up to 15 stalls | 305 | 10 (3.3) | 0.64 (0.20-2.08) | 0.457 |
|  | More than 15 stalls | 390 | 17 (4.4) | 1.00 |  |
| **Use of disinfectant at LBM** | |  |  |  |  |
|  | Use only water or detergent | 519 | 22 (4.2) | 1.27 (0.32-5.02) | 0.729 |
|  | Use disinfectant | 176 | 5 (2.8) | 1.00 |  |
| **Selling geese** | |  |  |  |  |
|  | Yes | 245 | 11 (4.5) | 1.59 (0.47-5.39) | 0.457 |
|  | No | 450 | 16 (3.6) | 1.00 |  |
| **Selling non-poultry birds** | |  |  |  |  |
|  | Yes | 500 | 22 (4.4) | 2.06 (0.54-7.86) | 0.290 |
|  | No | 195 | 5 (2.6) | 1.00 |  |
| **Presence of dead birds on market ground (Observation)**^‡^ | |  |  |  |  |
|  | Yes | 80 | 3 (3.8) | 1.46 (0.23-9.23) | 0.685 |
|  | No | 615 | 24 (3.9) | 1.00 |  |
| **Air sample RT-PCR result for influenza A by M gene** | |  |  |  |  |
|  | Positive | 432 | 12 (2.8) | 0.50 (0.16-1.51) | 0.219 |
|  | Negative | 263 | 15 (5.7) | 1.00 |  |
| **Air sample RT-PCR result for A(H5) or A(non-subtypeable)** | |  |  |  |  |
|  | Positive result for A(H5) or A(non-subtypeable) | 259 | 3 (1.16) | 0.22 (0.06-0.88) | 0.032 |
|  | Negative | 436 | 24 (5.50) | 1.00 |  |

^†^ORs were adjusted for age and random effect at market level.

^‡^Research team observed by themselves during the survey period

During the baseline survey, among 695 participants with serum specimens, 27 workers (3.9%, 95% CI 2.7-5.6%) had an influenza A(H5N1) neutralizing antibody titer of 1:10. No exposures were associated with an influenza A(H5N1) neutralizing antibody titer of 1:10 except for working in a LBM where an air sample RT-PCR result was found positive for influenza A(H5) or A(unsubtypeable) (OR=0.22, 95% CI 0.06-0.88) (**Supplementary Table 3**).

**Supplementary Table 4**: Stall level characteristics of workers with a neutralizing antibody titer to influenza A(H5N1) virus of 1:10 by microneutralization assay (N=695)

|  | | All | A(H5N1) | OR^†^ (95% CI) | p-value | aOR^‡^ (95% CI) | p-value^§^ |
| --- | --- | --- | --- | --- | --- | --- | --- |
|  |  |  | Titer 1:10  n (%) |  |  |  |  |
| **All** | | 695 | 27 (3.9) |  |  |  |  |
| **Sell duck/geese** | |  |  |  |  |  |  |
|  | Yes | 74 | 5 (6.8) | 3.26 (0.99-10.73) | 0.052 | 3.77 (1.09-12.10) | 0.036 |
|  | No | 621 | 22 (3.5) | 1.00 |  |  |  |
| **Defeathering machine with cover (observed)** Whether use defeathering machine in the stall and cover it during defeathering process | |  |  |  |  |  |  |
|  | Used defeathering machine and cover it | 254 | 14 (5.5) | 1.37 (0.57-3.29) | 0.487 | 1.52 (0.62-3.74) | 0.362 |
|  | Used defeathering machine but didn’t cover it | 32 | 1 (3.1) | 0.82 (0.86-7.91) |  | 0.89 (.09-8.57) |  |
|  | Did not use defeathering machine | 409 | 12 (2.9) | 1.00 |  | 1.00 |  |
| **Poultry boiled before defeathering** | |  |  |  |  |  |  |
|  | Yes | 318 | 17 (5.3) | 1.53 (0.63-3.67) | 0.346 | 1.67 (0.69-4.06) | 0.260 |
|  | No | 377 | 10 (2.7) | 1.00 |  | 1.00 |  |
| **Are there separate slaughtering and selling areas in the stall (n missing=1)** | |  |  |  |  |  |  |
|  | Yes | 424 | 18 (4.2) | 1.31 (0.56-3.07) | 0.538 | 1.38 (0.59-3.28) | 0.459 |
|  | No | 270 | 9 (3.3) | 1.00 |  | 1.00 |  |
| **Remove birds during cleaning of poultry cages/areas (n missing=7)** | |  |  |  |  |  |  |
|  | Yes | 289 | 15 (5.2) | 2.24 (0.92-5.50) | 0.077 | 2.32 (0.94-5.71) | 0.067 |
|  | No | 399 | 10 (2.5) | 1.00 |  |  |  |
|  |  |  |  |  |  |  |  |

^†^ORs were adjusted for age and market-level clustering using a random-effect.

^‡^aORs were adjusted for age, presence of duck in the LBM, type and size of the LBM and market-level clustering using a random-effect.

^§^Test for trend if ordered categorical variable

At the stall level no exposures were associated with a neutralizing antibody titer to influenza A(H5N1) virus of 1:10 except for working in a stall where ducks or geese were sold (aOR=3.77, 95% CI 1.09-12.10) (**Supplementary Table 4**).

**Supplementary Table 5**: Individual level characteristics of workers with a neutralizing antibody titer to influenza A(H5N1) virus of 1:10 by microneutralization assay (N=695)

|  | | | All | Number with a neutralizing antibody titer to A(H5N1) virus of 1:10 (%) | OR^†^ (95% CI) | p-value | aOR^‡^ (95% CI) | p-value^§^ |
| --- | --- | --- | --- | --- | --- | --- | --- | --- |
|  |  |  |  |  |  |  |  |  |
| **All** | | | 695 | 27 |  |  |  |  |
| **Age (years), median (IQR)** | | | 29 (22,38) | 25 (20,27) | 0.95 (0.90-0.99) | 0.019 | 0.95 (0.90-0.99) | 0.017 |
| **Current smoker** | | |  |  |  |  |  |  |
|  | Yes | | 307 | 9 (2.9) | 0.62 (0.26-1.47) | 0.276 | 0.63 (0.26-1.48) | 0.288 |
|  | No | | 388 | 18 (4.6) | 1.00 |  | 1.00 |  |
| **No of years worked in the market, median (IQR)** | | | 8 (3,15) | 6 (4,10) | 1.00 (0.93-1.07) | 0.943 | 1.00 (0.92-1.07) | 0.916 |
| **No of years worked in the market** | | |  |  |  |  |  |  |
| <10 years | | | 354 | 19 (5.4) | 1.00 |  | 1.00 |  |
| ≥10 years | | | 341 | 8 (2.4) | 0.71 (0.25-1.98) | 0.510 | 0.70 (0.25-1.95) | 0.495 |
| **Average working hours, mean (SD)** | | | 12.2 (2.4) | 12.3 (2.4) | 0.97 (0.81-1.16) | 0.715 |  |  |
| **Average working hours** | | |  |  |  |  |  |  |
| Up to 8 hours | | | 63 | 2 (3.2) | 1.00 |  | 1.00 |  |
| >8 hours | | | 632 | 25 (4.0) | 0.82 (0.17-3.91) | 0.804 | 0.85 (0.18-4.05) | 0.834 |
| **Poultry care** | | |  |  |  |  |  |  |
|  | Yes | | 684 | 25 (3.7) | 0.13 (0.19-0.91) | 0.039 | 0.13 (0.02-0.91) | 0.040 |
|  | No | | 11 | 2 (18.2) | 1.00 |  | 1.00 |  |
|  | **1: Carrying poultry** | |  |  |  |  |  |  |
|  |  | Yes, with hands and/or baskets | 459 | 14 (3.1) | 0.51 (0.21-1.20) | 0.123 | 0.48 (0.20-1.15) | 0.101 |
|  |  | No | 236 | 13 (5.5) | 1.00 |  |  |  |
|  | **2: Feeding poultry** | |  |  |  |  |  |  |
|  |  | Yes | 619 | 25 (4.0) | 1.15 (0.25-5.42) | 0.857 | 1.14 (0.24-5.42) | 0.864 |
|  |  | No | 76 | 2 (2.6) | 1.00 |  |  |  |
|  | **3: Selling poultry** | |  |  |  |  |  |  |
|  |  | Yes | 666 | 23 (3.5) | 0.16 (0.40-0.62) | 0.008 | 0.16 (0.04-0.62) | 0.008 |
|  |  | No | 28 | 4 (14.3) | 1.00 |  | 1.00 |  |
| **Poultry slaughtering and processing** | | |  |  |  |  |  |  |
|  | Yes | | 650 | 27 (4.15) | 2.75 (0.47-∞)*** |  |  |  |
|  | No | | 45 | 0 (0) | 1.00 |  |  |  |
|  | **Slaughtering** | |  |  |  |  |  |  |
|  |  | Yes | 648 | 27 (4.2) | 2.83 (0.50-∞)*** |  |  |  |
|  |  | No | 47 | 0 (0) | 1.00 |  |  |  |
|  | **Scalding/boiling** | |  |  |  |  |  |  |
|  |  | Yes | 282 | 15 (5.3) | 1.22 (0.51-2.92) | 0.649 | 1.31 (0.54-3.16) | 0.548 |
|  |  | No | 413 | 12 (2.9) | 1.00 |  | 1.00 |  |
|  | **Defeathering (verbal report)** | |  |  |  |  |  |  |
|  |  | Yes, with hands | 314 | 9 (2.9) | 1.15 (0.22-5.94) | 0.778 | 1.12 (0.22-5.81) | 0.699 |
|  |  | Yes, with machine (+ hands) | 272 | 16 (5.9) | 1.67 (0.33-8.54) |  | 1.81 (0.35-9.27) |  |
|  |  | No | 109 | 2 (1.8) | 1.00 |  | 1.00 |  |
|  | **Eviscerating** | |  |  |  |  |  |  |
|  |  | Yes | 583 | 25 (4.3) | 1.46 (0.30-7.01) | 0.636 | 1.48 (0.31-7.13) | 0.623 |
|  |  | No | 112 | 2 (1.8) | 1.00 |  | 1.00 |  |
|  | **Washing processed poultry** | |  |  |  |  |  |  |
|  |  | Yes | 344 | 14 (4.1) | 0.91 (0.39-2.10) | 0.817 | 0.94 (0.40-2.19) | 0.880 |
|  |  | No | 351 | 13 (3.7) | 1.00 |  | 1.00 |  |
| **Cleaning related activities** | | |  |  |  |  |  |  |
|  | Yes | | 620 | 26 (4.2) | 2.10 (0.26-16.95) | 0.486 | 2.11 (0.26-17.03) | 0.485 |
|  | No | | 75 | 1 (1.33) | 1.00 |  | 1.00 |  |
|  | **Cleaning feeding tray/water container** | |  |  |  |  |  |  |
|  |  | Yes | 562 | 23 (4.1) | 1.01 (0.31-3.25) | 0.987 | 1.01 (0.31-3.27) | 0.985 |
|  |  | No | 133 | 4 (3.0) | 1.00 |  | 1.00 |  |
|  | **Collecting/transporting carcasses and offal** | |  |  |  |  |  |  |
|  |  | Yes | 558 | 25 (4.5) | 2.05 (0.44-9.55) | 0.357 | 2.00 (0.43-9.35) | 0.377 |
|  |  | No | 137 | 2 (1.5) | 1.00 |  | 1.00 |  |
|  | **Collecting/transporting faces and feathers** | |  |  |  |  |  |  |
|  |  | Yes | 521 | 23 (4.4) | 1.29 (0.41-4.11) | 0.666 | 1.28 (0.40-4.10) | 0.675 |
|  |  | No | 174 | 4 (2.3) | 1.00 |  | 1.00 |  |
|  | **Cleaning poultry cages/baskets/keeping area** | |  |  |  |  |  |  |
|  |  | Yes | 536 | 21 (3.9) | 0.61 (0.22-1.72) | 0.351 | 0.59 (0.21-1.69) | 0.327 |
|  |  | No | 159 | 6 (3.8) | 1.00 |  |  |  |
| **RT-PCR results** | | |  |  |  |  |  |  |
|  | **Influenza A (excluding seasonal influenza)** | |  |  |  |  |  |  |
|  |  | Positive | 99 | 3 (3.0) | 0.81 (0.22-2.95) | 0.747 | 0.80 (0.21-2.96) | 0.734 |
|  |  | Negative | 596 | 24 (4.0) | 1.00 |  | 1.00 |  |
|  | **A(H5) or A(non-subtypeable)** | |  |  |  |  |  |  |
|  |  | Positive | 71 | 3 (4.2) | 1.15 (0.31-4.28) | 0.836 | 1.14 (0.30-4.31) | 0.851 |
|  |  | Negative | 624 | 24 (3.9) | 1.00 |  | 1.00 |  |

^†^ORs were adjusted for age and market-level clustering using a random-effect.

^‡^aORs were adjusted for age, presence of duck in the LBM, type and size of the LBM and market-level clustering using random-effects.

^§^ORs were calculated using exact logistic regression unadjusted for clustering.

At the individual level there was a suggestion of an inverse association of having a neutralizing antibody titer of 1:10 with increasing age. An inverse association was also seen with those caring for or selling poultry only (i.e. not poultry cleaning or slaughtering) (**Supplementary Table 4**). These were mostly older workers (median age 47.5 years, IQR 33.5-54.5 years vs 28 years, IQR 22-38 years; p<0.001) and stall owners or self-employed (n=22, 91.7% vs n=304, 43.3%; p<0.001).

Our study had age patterns consistent with an earlier study in 2009 of workers with an HPAI H5N1 virus where those with neutralizing antibody titer ≥1:20 were younger compared to workers with an antibody titer <1:20.(1) Among the 99 participants with any respiratory specimen that tested positive for influenza A by RT-PCR, excluding seasonal influenza A virus subtypes (n=4, one for H3 & three for H1), 3 (3.0%) participants had influenza A(H5N1) neutralizing antibody titers of 1:10. Among those who had either a respiratory specimen that was RT-PCR negative for Influenza A or positive for Influenza A but on subtyping was positive for seasonal influenza A virus (n=596), 24 (4.0%) also had titers of 1:10. There was no evidence of an association between having an influenza A positive respiratory specimen and an A(H5N1) virus neutralizing antibody titer of 1:10 nor when limited to those with influenza A(H5N1) or influenza A(non-subtypeable) detected by RT-PCR (**Supplementary Table 5**).

**Supplementary Table 6**. Influenza A(H5N1) neutralizing antibody titers at baseline and follow-up in LBM workers with respiratory specimens positive for A(H5), A(H9) or A (non-subtypeable) by RT-PCR at baseline (N=86)

| **RT-PCR Results** | **A(H5N1) neutralizing antibody titer at baseline** | **A(H5N1) neutralizing antibody titer at follow up** | **n/N (%)** |
| --- | --- | --- | --- |
| Influenza A positive excluding seasonal influenza A viruses  (N=86^†^) | 1:10 | 1:10 | 2/3 (66.7) |
|  |  | <1:10 | 1/3 (33.3) |
|  | <1:10 | 1:10 | 1/83 (1.2) |
|  |  | <1:10 | 82/83 (98.8) |

^†^Blood specimens were collected both at baseline and during follow-up from 89 LBM workers (with RT-PCR positive respiratory specimens for Influenza A during baseline survey), among them 3 were excluded for having respiratory specimens positive for seasonal influenza A viruses during the baseline survey

**Supplementary Table 7**: Primers and probes for RT-qPCR test for air samples

| **Subtype** | **Primer/**  **Probe** | **Sequence (5’ – 3’)** | **Reference** |
| --- | --- | --- | --- |
| **M** | Forward | 5′ AGATGAGYCTTCTAACCGAGGTCG 3’ | Heine et al., 2015(2) |
|  | Reverse 1 | 5′ TGCAAAAACATCYTCAAGTCTCTG 3’ |  |
|  | Reverse 2 | 5′ TGCAAACACATCYTCAAGTCTCTG 3’ |  |
|  | Reverse 3 | 5′ TGCAAAGACATCYTCAAGTCTCTG 3’ |  |
|  | Reverse 4 | 5′ TGCAAATACATCYTCAAGTCTCTG 3’ |  |
|  | Probe | 5′ FAM-TCAGGCCCCCTCAAAGCCGA-TAMRA-3’ |  |
| **H5** | Forward 1 | 5′ AAACAGAGAGGAAATAAGTGGAGTAAAATT 3’ | CSIRO |
|  | Forward 2 | 5′ ATGGCTCCTCGGRAACCC 3’ |  |
|  | Reverse 1 | 5′ AAAGATAGACCAGCTACCATGATTGC 3’ |  |
|  | Reverse 2 | 5′ TTYTCCACTATGTAAGACCATTCCG 3’ |  |
|  | Probe 1 | 5′ FAM-TCAACAGTGGCGAGTTCCCTAGCA-TAMRA-3’ |  |
|  | Probe 2 | 5′ FAM-ATGTGTGACGAATTCMT-TAMRA-3’ |  |
| **H7** | Forward | 5′ AYAGAATACAGATWGACCCAGT 3’ | Hoffmann et al., 2016 (3) |
|  | Reverse | 5′ TAGTGCACYGCATGTTTCCA 3’ |  |
|  | Probe | 5′ FAM-TGGTTTAGCTTCGGGGCATCATG -BHQ1-3’ |  |
| **H9** | Forward | 5′ ATGGGGTTTGCTGCC 3’ | Hoffmann et al., 2016 (3) |
|  | Reverse | 5′ ATATACAAATGTTGCAYCTGCA 3’ |  |
|  | Probe | 5′ FAM-TTCTGGGCYATGTCCAATGG -BHQ1-3’ |  |

**Supplementary Table 8**: Primers and probes for RT-PCR test for human respiratory samples

| **Type/Subtype** | **Fragment** | **Primer/Probe** | **Sequence** |
| --- | --- | --- | --- |
| Influenza A | Matrix (M) | Forward Primer | CCMAGGTCGAAACGTAYGTTCTCTCTCTATC |
|  | Matrix (M) | Reverse Primer | TGACAGRATYTTTCTTGTCTTTAGCCAYTCCA |
|  | Matrix (M) | Probe | 5’(FAM)-ATYTCGGCTTTGAGGGGGGCCTG- (MGB)-3’ |
| H1pdm09 | HA | Forward Primer | AGAAAAGAATGTAACAGTAACACATCCTGT |
|  | HA | Reverse Primer | TGTTTCCACAATGTARGACCAT |
|  | HA | Probe | 5’(FAM)-CAGCCAGCAATRTTRCATTTACC- (MGB)-3’ |
| H3 | HA | Forward Primer | CTATTGGACAATAGTAAAACCGGGRGA |
|  | HA | Reverse Primer | GTCATTGGGRATGCTTCCATTTGG |
|  | HA | Probe | 5’(FAM)-AAGTAAACCCCKAGGAGCAATTAG- (MGB)-3’ |
| H5 | HA | Forward Primer | CGATCTAGAYGGGGTGAARCCTC |
|  | HA | Reverse Primer | CCTTCTCCACTATGTANGACCATTC |
|  | HA | Probe | 5’(FAM)-AGCCATCCCGCAACAACTACA- (MGB)-3’ |
| H7 | HA | Forward Primer | TGTGAYGAYGATTGYATGGCCAG |
|  | HA | Reverse Primer | ACATGATGCCCCGAAGCTAAAC |
|  | HA | Probe | 5’(FAM)-ATCTGTATTCTATTTTGCATTGCYTC- (MGB)-3’ |
| H9 | HA | Forward Primer | AATGTYCCTGTGACACATGCCAAAGA |
|  | HA | Reverse Primer | AGRTCACAAGAAGGRTTGCCATA |
|  | HA | Probe | 5’(FAM)-CATYCCATTRTGCTCTGTGTGGAG- (MGB)-3’ |

**References:**

1. Nasreen S, Uddin Khan S, Azziz-Baumgartner E, Hancock K, Veguilla V, Wang D, et al. Seroprevalence of antibodies against highly pathogenic avian influenza A (H5N1) virus among poultry workers in Bangladesh, 2009. PloS one. 2013;8(9):e73200.

2. Heine HG, Foord AJ, Wang J, Valdeter S, Walker S, Morrissy C, et al. Detection of highly pathogenic zoonotic influenza virus H5N6 by reverse-transcriptase quantitative polymerase chain reaction. Virology journal. 2015;12(1):18.

3. Hoffmann B, Hoffmann D, Henritzi D, Beer M, Harder TC. Riems influenza a typing array (RITA): An RT-qPCR-based low density array for subtyping avian and mammalian influenza a viruses. Scientific reports. 2016;6:27211.
